# High-Precision Indoor Visible Light Positioning Using Modified Momentum Back Propagation Neural Network with Sparse Training Point

**DOI:** 10.3390/s19102324

**Published:** 2019-05-20

**Authors:** Haiqi Zhang, Jiahe Cui, Lihui Feng, Aiying Yang, Huichao Lv, Bo Lin, Heqing Huang

**Affiliations:** 1 Key Laboratory of Photonics Information Technology, Ministry of Industry and Information Technology, Beijing 100081, China; bitzhanghaiqi@sina.com (H.Z.); jiahe.cui@eng.ox.ac.uk (J.C.); yangaiying@bit.edu.cn (A.Y.); oncepursuit@gmail.com (H.L.); 2 School of Optoelectronics, Beijing Institute of Technology, Beijing 100081, China; 3 China Academy of Electronics and Information Technology, Beijing 100041, China; huangheqingbit@163.com

**Keywords:** neural network, indoor visible light positioning, high accuracy, LED

## Abstract

In this letter, we propose an indoor visible light positioning technique using a Modified Momentum Back-Propagation (MMBP) algorithm based on received signal strength (RSS) with sparse training data set. Unlike other neural network algorithms that require a large number of training data points to locate accurately, we have realized high-precision positioning for 100 test points with only 20 training points in a 1.8 m × 1.8 m × 2.1 m localization area. In order to verify the adaptability of the MMBP algorithm, we experimentally demonstrate two different training data acquisition methods adopting either even or arbitrary training sets. In addition, we also demonstrate the positioning accuracy of the traditional RSS algorithm. Experimental results show that the average localization accuracy optimized by our proposed algorithm is only 1.88 cm for the arbitrary set and 1.99 cm for the even set, while the average positioning error of the traditional RSS algorithm reaches 14.34 cm. Comparison indicates that the positioning accuracy of our proposed algorithm is 7.6 times higher. Results also show that the performance of our system is higher than some previous reports based on RSS and RSS fingerprint databases using complex machine learning algorithms trained by a large amount of training points.

## 1. Introduction

In recent years, with the rapid development of wireless sensor networks and the Internet of Things, indoor positioning technology has been used in many fields, such as shopping guidance, intelligent robots, and so on. Traditional positioning techniques based on radio wave communication, such as the extensively used global positioning system (GPS), suffer from multipath fading and severe attenuation, which lead to large positioning errors in the indoor environment. Other indoor positioning systems such as wireless local area network (WLAN), ZigBee, Ultra-Wideband (UWB), Bluetooth, and Radio-frequency Identification (RFID) deliver positioning errors from tens of centimeters to a few meters [1,2]. Visible light communication (VLC) systems based on LEDs have emerged in recent years and show many advantages. Firstly, LEDs are cost-effective, energy-efficient and widely utilized for indoor illumination. Secondly, VLC systems have a strong ability to resist electromagnetic interference [3,4]. 

For the above reasons, more and more researchers are focusing on how to improve the performance of indoor visible light positioning (VLP) systems. Both photo-diodes (PDs) and image sensors can be utilized as the detector [5,6,7,8,9,10,11]. Taking into consideration the system complexity and cost, PD-based systems using the RSS algorithm are most widely reported [5,6,7,8]. In 2012, W. Zhang et al. proposed a PD-based visible light positioning system to locate a target within centimeters in the simulation [5]. In [6], the average distance error was 1.7 cm and 2.1 cm in the *x*- and *y*-coordinates within a space of 94.8 cm × 80 cm × 127.7 cm based on RSS. In addition, typical PD-based algorithms are more practical in large scenarios where more objects need to be located [7]. 

As known to all, the positioning error performance of VLP based on RSS is usually poor, so researchers have begun to exploit new methods to scale the accuracy based on RSS. In recent works, machine learning has been used in VLP based on RSS. In [12], the average positioning error was only 6.39 cm in the simulation stage with the Levenberg-Marquardt algorithm and the number of training data was 2630 within a 5 m × 5 m × 3 m area. Earlier this year, Hsu et al. realized 3.65 cm accuracy in a 1 m × 0.9 m triangular unit cell [13]. However, this learning method was very slow and the parameters in this paper were corrected iteratively with 5680 training points. In addition, combining an RSS fingerprint database with machine learning is also reported in some papers. In [14], Zhang. C proposed a lightweight fingerprint-based indoor positioning algorithm using generalized regression neural network (GRNN) to approach the time-consuming and labor-intensive process of fingerprint surveying. They selected 140 training points in the area of 3 m × 5 m and the average positioning error was 8.7 cm. In [15], Guo. X proposed a localization technique by fusing multiple classifiers based on RSS fingerprint base of visible light and realized high precision indoor positioning. However, the experiment was only performed within a 0.7 m × 0.7 m area. In [16], Guo X proposed a two-layer fusion network (TLFN) indoor localization method using the same visible light positioning scenario as [15] and the average positioning error was 5 cm. 

In our paper, we mainly focus on the application of machine learning algorithms in a 2D localization scene and how to achieve high-precision positioning results with sparse training points. Therefore, the height of the detector isn’t our main consideration when collecting the training data. In this letter, a VLP system based on RSS and modified momentum back-propagation (MMBP) artificial neural network (ANN) is implemented under a large experimental environment. The ANN is trained using the MMBP method. Unknown positions across the whole positioning area can then be calculated using the trained ANN. Experimental results show that the average localization accuracy using the arbitrary training set is 1.88 cm in a 1.8 m × 1.8 m × 2.1 m cell with 1.42 cm and 0.88 cm along the *x*- and *y*-axis respectively, and the maximum positioning error is only 5 cm. For the even set, the average localization accuracy is 1.99 cm with 1.49 cm and 0.83 cm in the *x*- and *y*-axis, while bringing a 9.64 cm maximum positioning error. In comparison with the traditional RSS location algorithm, our positioning accuracy is 7.6 times higher. Furthermore, compared with the results based on RSS fingerprint in [15], our results are more accurate and the size of the training set is only 1/11 of that reported in Reference [11]. 

## 2. Theory and Methods

**Figure 1:**
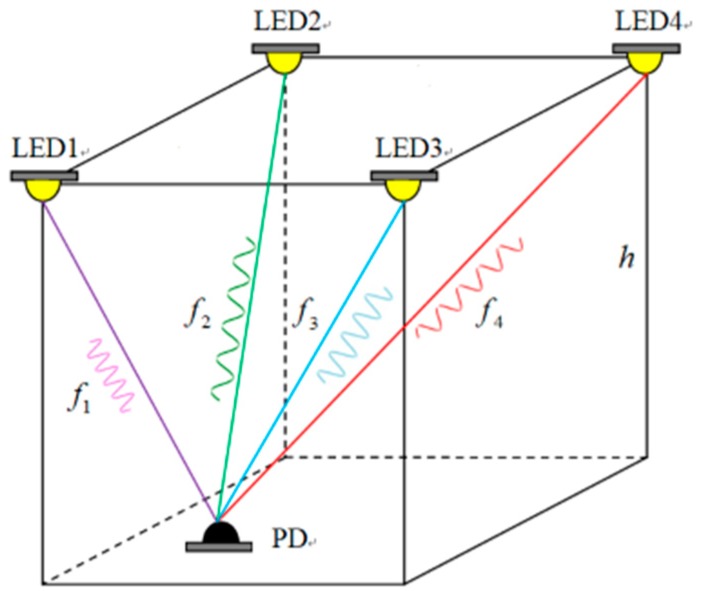
Typical indoor visible light positioning (VLP) scenario with modulated LEDs.

In our proposed system, a typical indoor scenario with four LEDs and a photo-detector is investigated, as shown in Figure 1. To overcome the signal interference between densely arranged LEDs, we have allocated a unique modulation frequency to each of the four LEDs. The shape of a room is normally rectangular or square, a positioning system composed of four LEDs is nearest to the actual positioning scene. In addition, considering cost and complexity, we believe our positioning system based on four LEDs is the most suitable. Using more than four LEDs would mean higher complexity and cost. If the number of LED in the positioning area is more than 4, we can achieve the positioning by selecting four LEDs as the inter-cell in the area.

The RSS information which contains four different frequencies is detected by the detector and extracted by Fourier transformation, then used as inputs of the network. The output of our network is the position of the detector. The frequency of background light is different from the transmitted signal and can be ignored during demodulation. Supposing that the LEDs have a Lambertian radiation pattern, when only the line-of-sight (LOS) channel is considered, the distance between an LED and the receiver can be estimated by measuring the received signal power, which can be expressed by [12].

(1) $$P_{r}^{i}=\frac{A_{r}\left(m+1\right)}{2\pi d_{i}{}^{2}}\cos^{m}(\phi_{i})\cos(\varphi_{i})T_{trans}g\left(\varphi_{i}\right)P_{t}{}_{i},i=1,2,3,4$$

where $P_{r}^{i}\left(i=1,2,3,4\right)$ is the received signal power of the four LEDs, $P_{t}$ is the transmitted signal power, $A_{r}$ is the physical area of the detector, d is the distance between the LED and the receiver, ϕ is the angle of irradiance, φ is the angle of incidence, $T_{trans}$ is the optical transmittance, g(φ) is the gain of the optical concentrator and m is the Lambertian mode number related to the semi-angle at half-power $\phi_{1/2}$ of the LED, denoted by.

(2) $$m=\frac{\ln(2)}{\ln(\cos\phi_{1/2})}$$

### 2.1. Traditional RSS Algorithm

Traditional RSS-based trilateration algorithms adopt the radio frequency allocation (RFA) technique. The signals transmitted from various LEDs are detected by a detector and distinguished according to their frequencies. In the scenario, we set the LED coordinate as $\left(x_{1},y_{1},z\right),\;\;\left(x_{2},y_{2},z\right),\;\;\left(x_{3},y_{3},z\right)$, and $\left(x_{4},y_{4},z\right)$. The height of detector is $h_{s}$. In our case, the detector is placed on the ground, therefore $h_{s}=0\;$m. In the experiment, the position of the detector is (x,y) and it needs to be calculated by the algorithm. The transmitted power of LED 1, LED 2, LED 3, and LED 4 are denoted as $P_{t1},\;P_{t2},\;P_{t3},\;P_{t4}$ and the received signal strengths as $P_{r1},\;P_{r2},\;P_{r3},\;P_{r4}$, respectively. According to Equation (1), we have

(3) $$\{\begin{array}{c} \frac{P_{r1}}{P_{t1}}=H_{1}\left(0\right)=\frac{A_{r}\left(m+1\right)}{2\pi d_{1}^{2}}\cos^{m}(\phi_{1})\cos(\varphi_{1})T_{trans}g\left(\varphi_{1}\right)\\ \frac{P_{r2}}{P_{t2}}=H_{2}\left(0\right)=\frac{A_{r}\left(m+1\right)}{2\pi d_{2}^{2}}\cos^{m}(\phi_{2})\cos(\varphi_{2})T_{trans}g\left(\varphi_{2}\right)\\ \frac{P_{r3}}{P_{t3}}=H_{3}\left(0\right)=\frac{A_{r}\left(m+1\right)}{2\pi d_{3}^{2}}\cos^{m}(\phi_{3})\cos(\varphi_{3})T_{trans}g\left(\varphi_{3}\right)\\ \frac{P_{r4}}{P_{t4}}=H_{4}\left(0\right)=\frac{A_{r}\left(m+1\right)}{2\pi d_{4}^{2}}\cos^{m}(\phi_{4})\cos(\varphi_{4})T_{trans}g\left(\varphi_{4}\right) \end{array}$$

In our experiment, $A_{r}$ can be obtained from the detector datasheet, m can be calculated by Equation (2) and the semi-angle at half-power $\phi_{1/2}$ can be obtained from the LED datasheet. $T_{trans}$ and g(φ) can be considered as constants. The detector plane is placed parallel to the floor. Then $\phi_{i}$ and $\varphi_{i}$ can be expressed as

(4) $$\{\begin{array}{c} \cos(\phi_{i})=\left(z-h_{s}\right)/d_{i}\\ \cos(\varphi_{i})=\left(z-h_{s}\right)/d_{i} \end{array}$$

And Equation (3) can be expressed as

(5) $$\{\begin{array}{c} \frac{P_{r1}}{P_{t1}}=H_{1}\left(0\right)=\frac{C}{d_{1}^{m+3}}\\ \frac{P_{r2}}{P_{t2}}=H_{2}\left(0\right)=\frac{C}{d_{2}^{m+3}}\\ \frac{P_{r3}}{P_{t3}}=H_{3}\left(0\right)=\frac{C}{d_{3}^{m+3}}\\ \frac{P_{r4}}{P_{t4}}=H_{4}\left(0\right)=\frac{C}{d_{4}^{m+3}} \end{array}$$

where C is the parameter related to the hardware of the VLP and can be expressed by

(6) $$C=\frac{A_{r}\left(m+1\right){\left(z-h_{s}\right)}^{m+1}}{2\pi }T_{trans}g\left(\varphi \right)$$

where g(φ) is a constant with the same value in all directions within the field of view and therefore we have $g\left(\varphi \right)=g\left(\varphi_{1}\right)=g\left(\varphi_{2}\right)=g\left(\varphi_{3}\right)=g\left(\varphi_{4}\right)$ in Equation (3). According to the relationship between the detector and four LEDs in the positioning area, we get

(7) $$\{\begin{array}{c} {\left(x-x_{1}\right)}^{2}+{\left(y-y_{1}\right)}^{2}+{\left(z-h_{s}\right)}^{2}=d_{1}^{2}\\ {\left(x-x_{2}\right)}^{2}+{\left(y-y_{2}\right)}^{2}+{\left(z-h_{s}\right)}^{2}=d_{2}^{2}\\ {\left(x-x_{3}\right)}^{2}+{\left(y-y_{3}\right)}^{2}+{\left(z-h_{s}\right)}^{2}=d_{3}^{2}\\ {\left(x-x_{4}\right)}^{2}+{\left(y-y_{4}\right)}^{2}+{\left(z-h_{s}\right)}^{2}=d_{4}^{2} \end{array}$$

Combining Equations (5) and (7), we get

(8) $$\{\begin{array}{c} {\left(x-x_{1}\right)}^{2}+{\left(y-y_{1}\right)}^{2}+{\left(z-h_{s}\right)}^{2}={\left(\frac{CP_{t1}}{P_{r1}}\right)}^{\frac{2}{m+3}}\\ {\left(x-x_{2}\right)}^{2}+{\left(y-y_{2}\right)}^{2}+{\left(z-h_{s}\right)}^{2}={\left(\frac{CP_{t2}}{P_{r2}}\right)}^{\frac{2}{m+3}}\\ {\left(x-x_{3}\right)}^{2}+{\left(y-y_{3}\right)}^{2}+{\left(z-h_{s}\right)}^{2}={\left(\frac{CP_{t3}}{P_{r3}}\right)}^{\frac{2}{m+3}}\\ {\left(x-x_{4}\right)}^{2}+{\left(y-y_{4}\right)}^{2}+{\left(z-h_{s}\right)}^{2}={\left(\frac{CP_{t4}}{P_{r4}}\right)}^{\frac{2}{m+3}} \end{array}$$

We can therefore attain the position of the detector by solving Equation (8). It should be noted that Equation set (8) is over-determined, and is therefore solved by the computer. However, in practical scenarios, the amount of power detected by the receiver can be affected by many external factors including multipath reflections and the incidence angle, leading to large positioning errors. 

### 2.2. Modified Momentum Back Propagation (MMBP) Algorithm 

**Figure 2:**
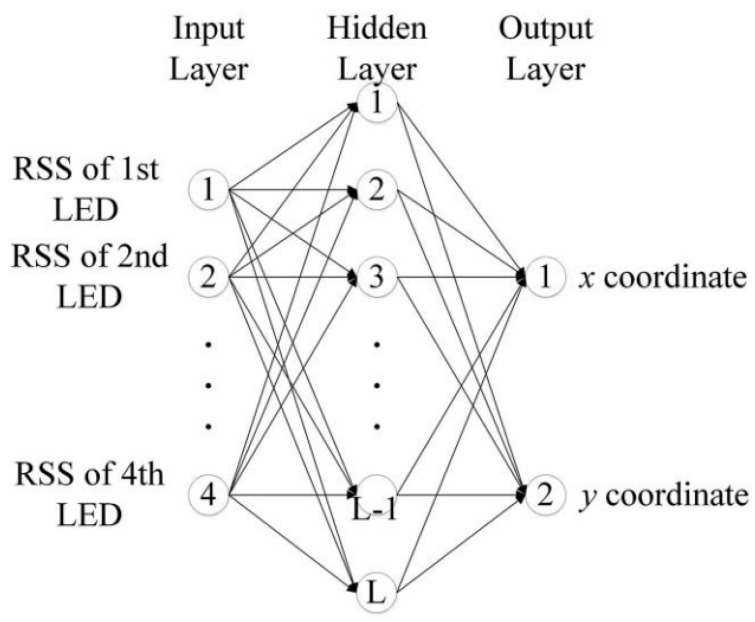
Structure of multi-layer feed-forward neural network.

The results shown in [12] prove the effectiveness of BP algorithms in reducing positioning errors and the verified positioning error is 6.39 cm. In fact, the main advantage of BP neural network is the outstanding ability of non-linear mapping and classification which is ideal for applications in indoor positioning systems [17]. Therefore, the BP algorithm is very suitable for indoor positioning due to the characteristics of indoor scenes [18,19,20,21]. In this paper, we use the Back-Propagation algorithm based on multi-layer feed-forward neural networks to realize high precision indoor positioning. The number of hidden layers is a critical factor determining the complexity of neural networks, and complex neural networks need a lot of training data to train. If we train complex neural networks with large amounts of training data, we would presumably achieve high-precision positioning results. However, as we mainly focus on the question of how to achieve high-accuracy positioning with sparse training points, the number of training data in our paper is only 20. Complex neural networks trained by sparse training data often lead to over-fitting. Therefore, to prevent over-fitting, we have selected only one hidden layer in the neural network and verified the effect of different numbers of hidden layers on the positioning accuracy. The structure of the multi-layer feed-forward neural network based on the proposed MMBP algorithm mainly consists of three parts, namely the input layer, hidden layer, and the output layer. The number of nodes in the hidden layer is determined through experiment and the output of our network is the position of the detector. The structure of multi-layer feed-forward neural network is shown in Figure 2:

We set the received signal strength from LED 1, LED 2, LED 3, LED 4 as the input of the neural network and it can be expressed by $x=\left(x_{1},\;x_{2},\;x_{3}\;\ldots x_{n}\right)$ where n=4 in this paper. The number of hidden layer nodes and output layer nodes are denoted as r and m, respectively. In this paper, the value of m is 2. The output of the neural network is the position of the detector and is expressed as h(x,y); $w_{ij}$ is the weight between the input layer and hidden layer and the threshold is $\theta_{j}$. $w_{jk}$ is the weight between the hidden layer and output layer and the threshold is $\theta_{k}$. Then we have the output of the hidden layer [22]:

(9) $$x_{j}=f\left(\overset{n}{\underset{i=1}{\sum }}w_{ij}x_{i}-\theta_{j}\right),\;j=1,\;2,\;3\;\ldots r$$

The output of the output layer is

(10) $$h\left(x_{j},y_{j}\right)=f\left(\overset{r}{\underset{j=1}{\sum }}w_{jk}x_{j}-\theta_{k}\right),k=1,\;2$$

When p  denotes the p th input of the neural network, $d^{p}$ and $y^{p}$ are the  p th desired output and real output, respectively. Then the  p th mean square error of the neural network can be expressed as

(11) $$E^{\left(p\right)}=\frac{1}{2}\overset{m}{\underset{l=1}{\sum }}{\left(d_{l}^{\left(p\right)}-y_{l}^{\left(p\right)}\right)}^{2}$$

And the total output error of the trained neural network is as follows:

(12) $$E_{A}=\overset{p}{\underset{p=1}{\sum }}E^{\left(p\right)}=\frac{1}{2}\overset{p}{\underset{p=1}{\sum }}\overset{m}{\underset{l=1}{\sum }}{\left(d_{l}^{\left(p\right)}-y_{l}^{\left(p\right)}\right)}^{2}$$

Let $w_{sp}$ be a connection weight in the network, then according to the gradient descent method, the weight correction should be as follows:

(13) $$\Delta w_{sp}=-\eta \frac{\partial E}{\partial w_{sp}}$$

Taking into consideration the complexity of practical environments, in order to increase the convergence rate of our network and avoid oscillation, in our algorithm, we have adopted a highly efficient learning method that combines momentum BP algorithm with variable learning rate BP algorithm. The update of weight coefficients between layers is guided by a momentum factor α so that

(14) $$\Delta \omega \left(n\right)=-\eta \left(1-\alpha \right)\frac{\partial E}{\partial w_{sp}\left(n-1\right)}+\alpha \Delta \omega \left(n-1\right)$$

where Δω(n) and Δω(n−1) are the correction values of weight coefficients in iteration n and n−1, η is the learning rate, and $\frac{\partial E}{\partial w_{sp}}$ is the error gradient of the network. The learning rate η self-adapts itself to the network decided by the direction in which the error changes and the update formula of η can be expressed by

(15) $$\eta \left(n+1\right)=\{\begin{array}{c} k_{inc}\eta \left(n\right),\;\;\frac{\partial E}{\partial w_{sp}\left(n\right)}<\frac{\partial E}{\partial w_{sp}\left(n-1\right)}\\ k_{dec}\eta \left(n\right),\;\;\frac{\partial E}{\partial w_{sp}\left(n\right)}>\frac{\partial E}{\partial w_{sp}\left(n-1\right)} \end{array}$$

where $k_{inc}$ is a constant increment factor and $k_{dec}$ is a constant decrement factor both determined by the actual performance of the network.

**Figure 3:**
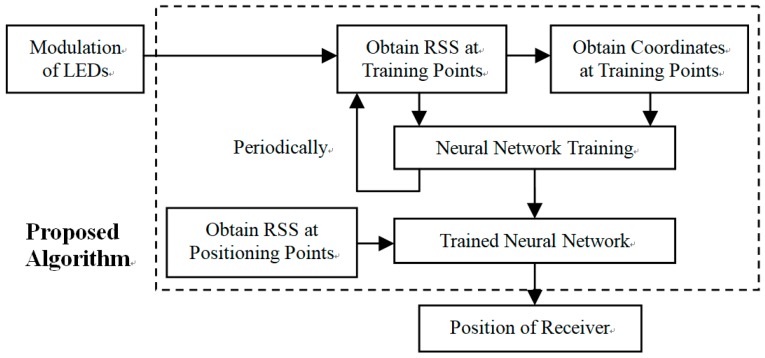
Flow chart of proposed Modified Momentum Back-Propagation (MMBP) algorithm.

Our ANN functions through two stages. In the training stage, the network is trained by the RSS values of a chosen set of training points in the positioning area. The total squared error between desired coordinates and the actual output of the network is optimized by the MMBP algorithm. If the training is completed, the neural network enters the testing stage. During this process, the input of the neural network is the position of the test points, and then the coordinates of test points can be calculated by the neural network. The flow chart of the ANN-based VLP algorithm is shown in Figure 3.

## 3. Experiment and Results

### 3.1. Experimental Facilities

**Figure 4:**
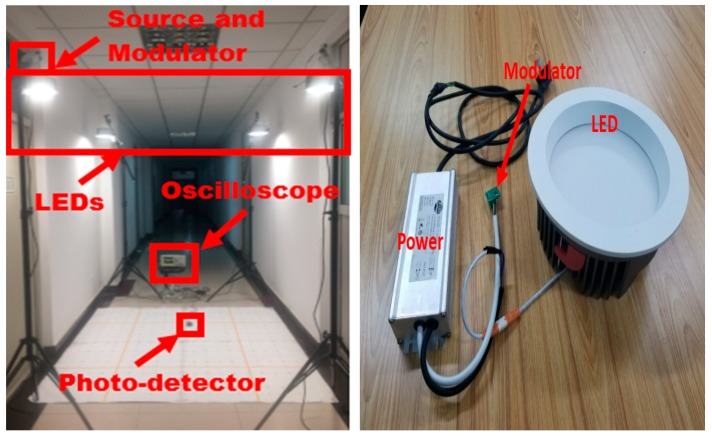
(**a**) Experimental setup of positioning system, (**b**) LED transmitter.

The effectiveness of the proposed BP Neural Network -based VLP system is verified under indoor environment. As in Figure 4a, four commercial LEDs that transmit different frequencies of 4100 Hz, 3000 Hz, 5000 Hz and 2200 Hz, are mounted on the ceiling of a 1.8 m × 1.8 m × 2.1 m positioning area. The locations of the four LEDs are (0, 0, 2.1), (0, 1.8, 2.1), (1.8, 0, 2.1), and (1.8, 1.8, 2.1), respectively. We have used a Thorlabs (APD110A2) as the detector and the transmitter is shown in Figure 4b. The radio frequency signal is modulated into the optical signal using a modulator based on the STM32 F103. Specific parameters of the positioning system are shown in Table 1.

In our experiment, 100 test points (blue points) are evenly placed in the positioning area with a density of 20 cm × 20 cm. Twenty points (red points) are selected as the training set, and all 100 test points are positioned in the validation stage. We perform measurements five times at each training data point (red point) to reduce the influence of light intensity fluctuation on the positioning error and save them as the training data set. In order to verify the adaptability of the proposed algorithm, we use two different training data sets: arbitrary set and even set. 

### 3.2. Result and Analysis

**Figure 5:**
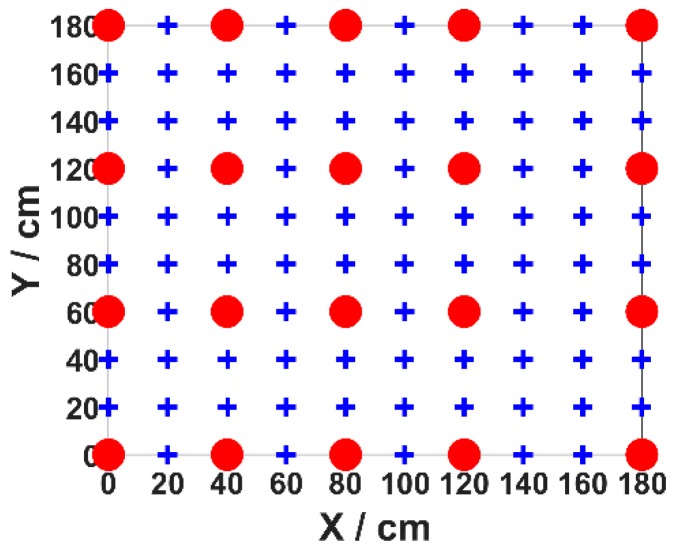
Even training data set.

**Figure 6:**
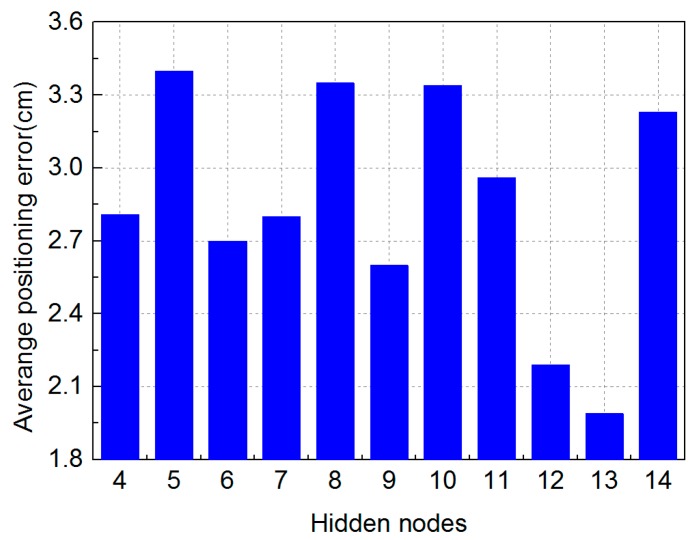
Positioning error with different numbers of hidden layer nodes under the even training data set.

In our experiment, we firstly demonstrate the positioning error optimized by MMBP algorithm with the even training data set and the even training data set is shown in Figure 5. The number of nodes in the hidden layer of the ANN is determined experimentally by evaluating its effect on positioning accuracy. We define the average positioning error as the same as in Reference [15]. Figure 6 shows the corresponding average positioning error with different numbers of hidden layer nodes.

Increasing the number of hidden layer nodes can reduce the positioning error, but at the cost of making the neural network more complex and leading to increased training time. We have therefore selected the number of hidden layer nodes from 4 to 14 due to the limited number of training points. The average positioning error with different numbers of nodes under the even training data set is shown in Figure 6. We can see that the best performance is obtained when there are 13 nodes in the hidden layer and the average positioning error is 1.99 cm, suggesting that a 13-neuron structure is most optimal for this scenario. Other parameters of the neural network using the even training set are shown in Table 2.

**Figure 7:**
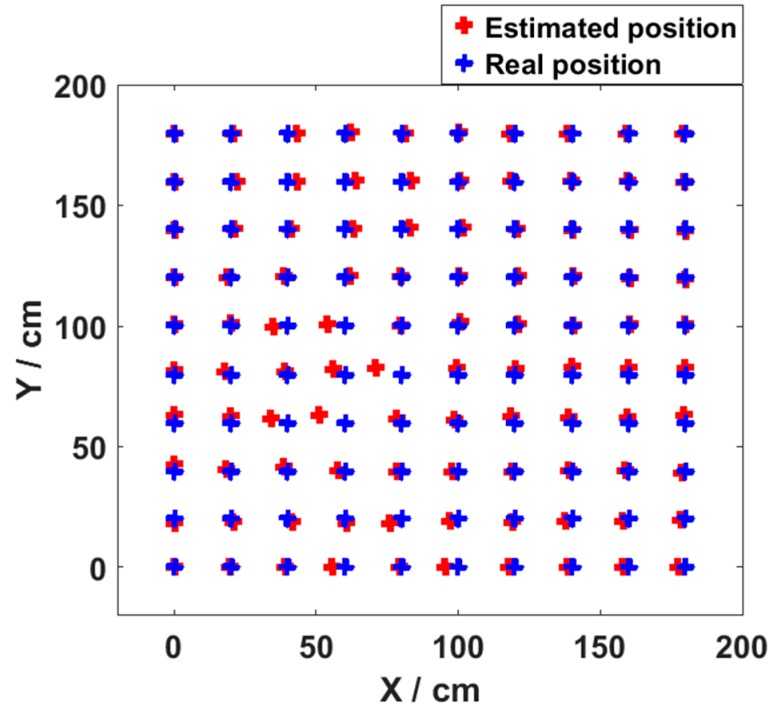
Final 2D positioning results with even training data set.

Meanwhile, in Figure 6, the maximum positioning error observed with different number of nodes is only 3.35 cm. This demonstrates the high positioning accuracy of the MMBP algorithm. The 2D positioning results in the validation stage at all 100 points are shown in Figure 7. It verifies that after being trained with only 20 points, the neural network is able to perform accurately for all other points in the positioning area, and the average positioning error is 1.99 cm.

Then we compare the average positioning error using the traditional RSS algorithm. We have derived the calculation process earlier in the paper, which can be referred to in Equations (1)–(8).

**Figure 8:**
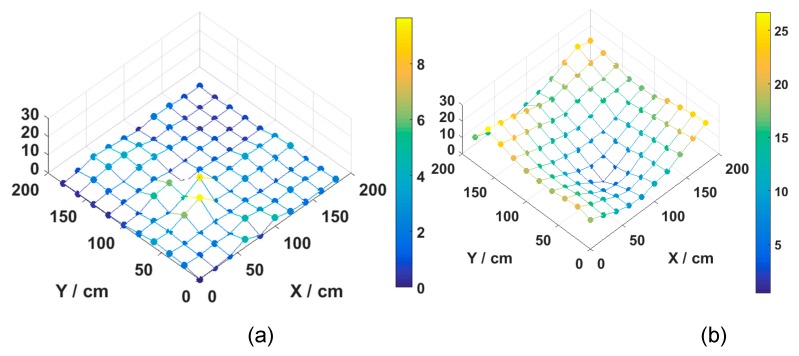
Distribution of positioning error for (**a**) MMBP algorithm with even training data set and (**b**) traditional received signal strength (RSS)-based algorithm.

**Figure 9:**
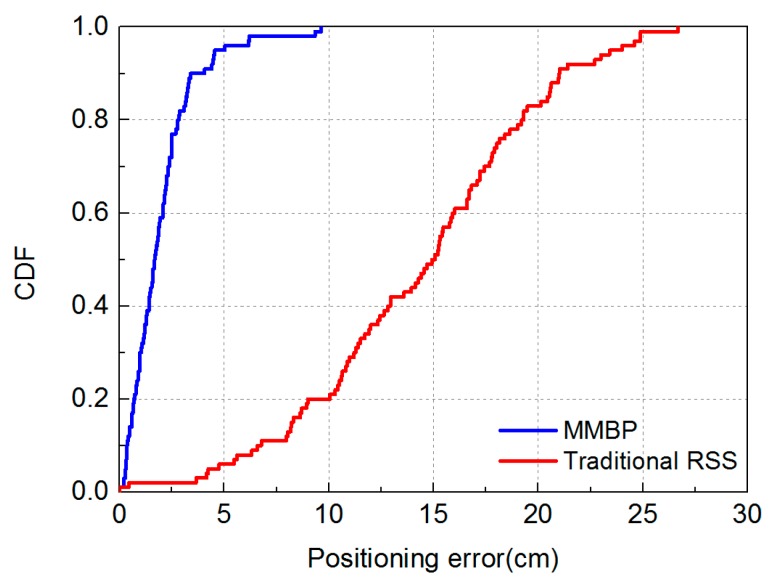
Cumulative distribution of positioning error for the MMBP algorithm using even training data set and traditional RSS-based algorithm.

**Figure 10:**
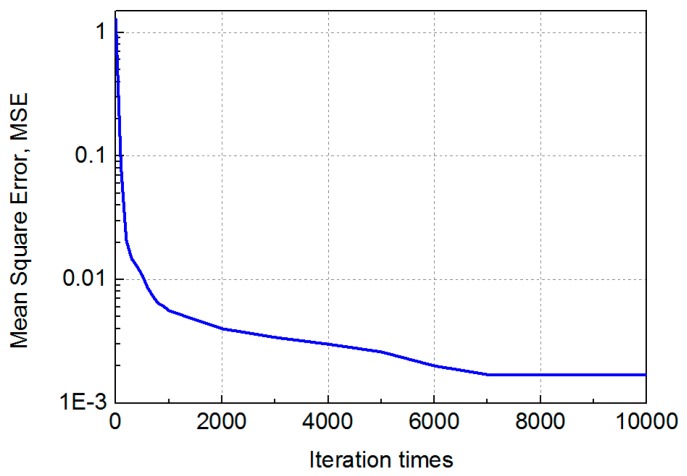
MSE performance with the even training data set.

We evaluate the effectiveness of MMBP by comparing its performance with the traditional RSS-based algorithm. The distribution of positioning error of MMBP and traditional RSS-based algorithm are shown in Figure 8a,b. The corresponding cumulative distribution function (CDF) of positioning errors is presented in Figure 9. Results indicate that the performance of MMBP is much better than the traditional algorithm, where the average estimation error is significantly lowered by more than one magnitude from 14.34 cm to 1.99 cm. The accuracy along the *x*-axis is improved from 10.14 cm to 1.49 cm while that along the *y*-axis is improved from 8.55 cm to 0.83 cm. Figure 9 shows that the maximum positioning error for the traditional RSS algorithm is 26.66 cm and the error of 80% confidence is 19.31 cm. In comparison, with the MMBP algorithm, the largest positioning error is successfully kept within 10 cm (9.64 cm), and the error of 80% confidence is reduced to 2.77 cm. The average positioning accuracy of the MMBP algorithm is 7.2 times higher than the traditional RSS algorithm. The MSE of the MMBP with the even training data set is shown in Figure 10. Although the MSE declined rapidly, we can see that the mean square error reaches the minimum value after 8000 iterations. In addition, in Table 3, the positioning time of traditional RSS-based algorithm is 2.25 s, while the positioning time of our proposed algorithm is only 0.007s and the training time is 2.36 s (CPU: AMD FX-6300). The comparison results prove that the proposed algorithm can achieve high-precision and real-time positioning.

**Figure 11:**
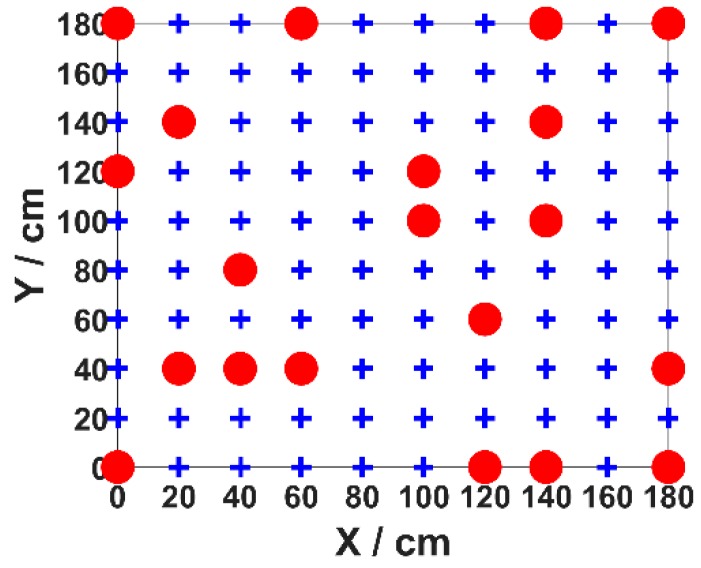
Arbitrary training data set.

**Figure 12:**
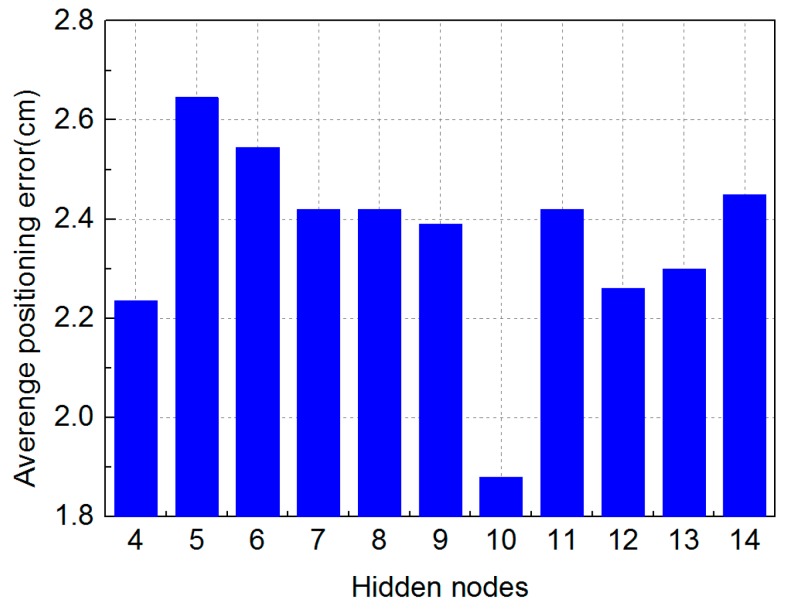
Positioning error with different numbers of hidden layer nodes under the arbitrary training data set.

To verify the adaptability of the proposed algorithm, we also demonstrate the performance achieved using an arbitrary training set and the arbitrary training data set is shown in Figure 11. Unlike the neural network with the even training data set, best performance is obtained with 10 nodes in the hidden layer. The experimental results are shown in Figure 12.

**Figure 13:**
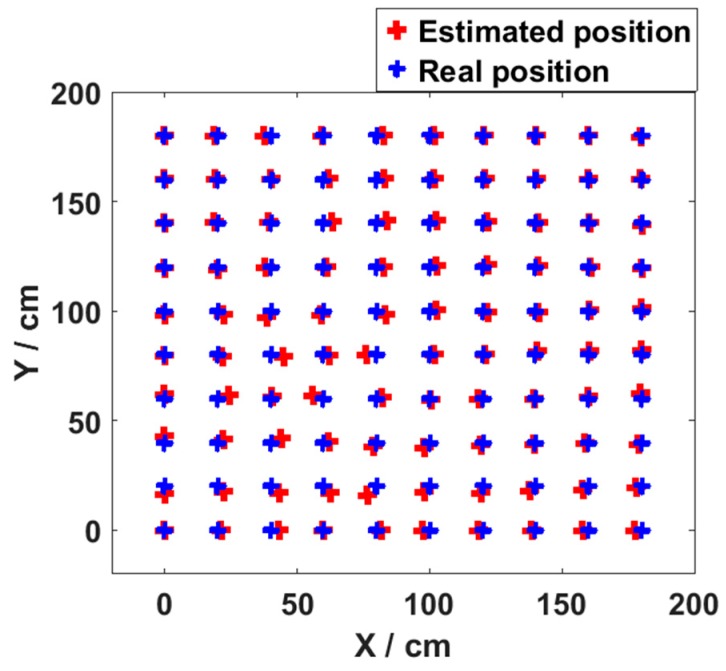
Final 2D positioning results with arbitrary training data set.

The parameters of the neural network with the arbitrary training data set are shown in Table 4. It can be seen from Figure 12 that the maximum average positioning error is only 2.64 cm which is obviously much lower than the results shown in Figure 6. Meanwhile, the minimum average positioning error is only 1.88 cm when the number of hidden layer nodes is 10. Comparing Figure 6 with Figure 12, we can find that the neural network trained by the arbitrary training data set has better positioning performance. The 2D positioning results in the validation stage at all 100 points are shown in Figure 13. We can see that after being trained with only 20 points, the network is able to perform accurately for all other points in the positioning area, and the average positioning error is 1.88 cm. 

**Figure 14:**
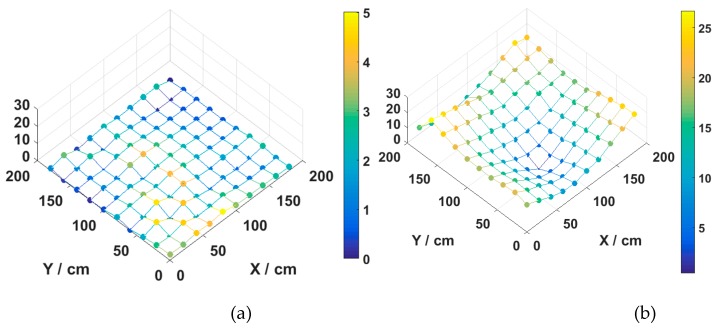
Distribution of positioning error for (**a**) MMBP algorithm with arbitrary training data set and (**b**) traditional RSS-based algorithm.

**Figure 15:**
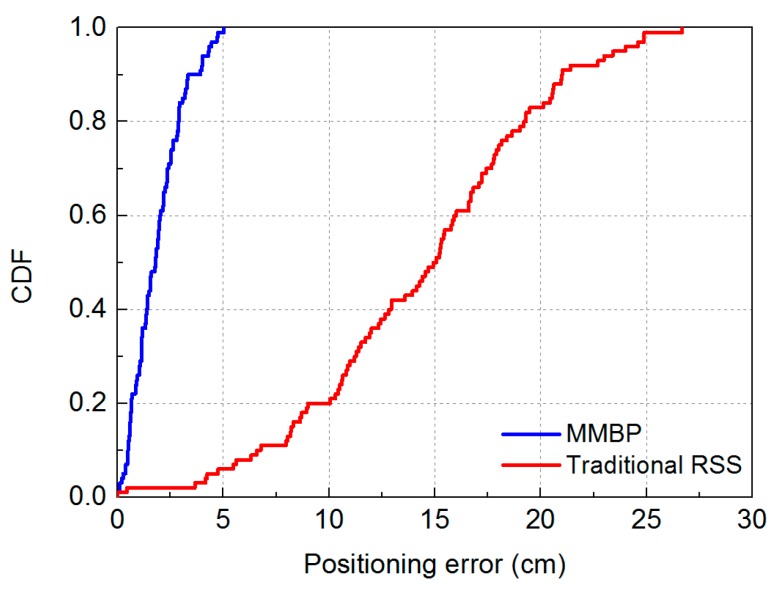
Cumulative distribution of positioning error for the MMBP algorithm using arbitrary training data set and traditional RSS-based algorithm.

The distribution of positioning error of MMBP algorithm and the traditional RSS-based algorithm are shown in Figure 14a,b. The maximum positioning error is 5 cm, which is just half as that obtained with the even training data set. In addition, the distribution of positioning errors with the even training data set is different from that of the arbitrary set due to different distributions of training points. The corresponding cumulative distribution function (CDF) of positioning errors is presented in Figure 15. 

**Figure 16:**
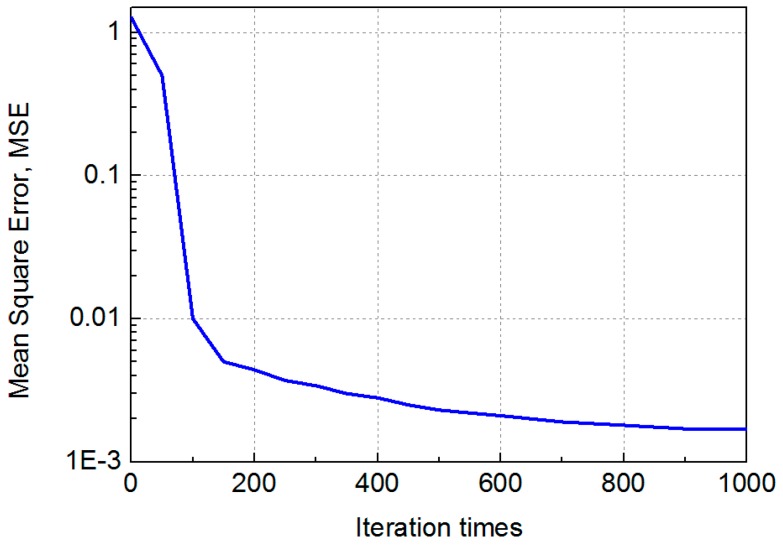
MSE performance with the arbitrary training data set.

Results shown in Figure 14 and Figure 15 indicate that the performance of the MMBP algorithm is much better than the traditional RSS algorithm, where the average estimation error is significantly lowered by more than one magnitude from 14.34 cm to 1.88 cm. The accuracy along the *x*-axis is improved from 10.14 cm to 1.42 cm while that along the *y*-axis is improved from 8.55 cm to 0.88 cm. Figure 15 shows that the maximum positioning error for the traditional RSS algorithm is 26.66 cm and the error of 80% confidence is 19.31 cm. In comparison, with our proposed algorithm, the largest positioning error is only 5 cm, and the error of 80% confidence is reduced to 2.88 cm. The average positioning accuracy of MMBP algorithm is 7.6 times higher than the traditional RSS algorithm. Figure 16 shows the MSE performance with the arbitrary training data set. Compared with the results shown in Figure 10, after only 900 iterations, the mean square error of the proposed algorithm reaches the minimum value. Then we test the time cost of the algorithm and the results are shown in Table 5. We can find the positioning and training time with the arbitrary data set are only 0.005 s and 0.403 s, respectively, which are faster than the results with the even data set due to fewer hidden layer nodes and iteration times.

**Figure 17:**
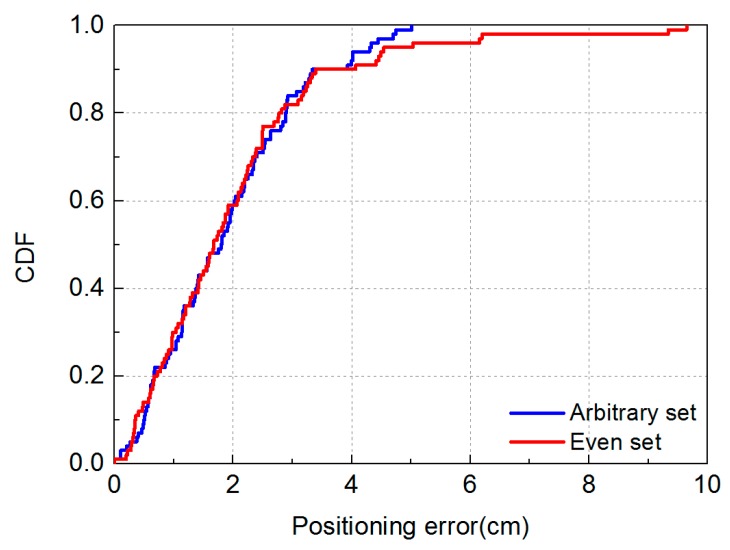
Cumulative distribution of positioning error for the arbitrary training data set and even training data set.

In Figure 17, we can find that the maximum positioning error is obviously different when the neural network is trained by different training data sets. The maximum positioning error for the arbitrary set is only 5 cm while that for the even set is 9.64 cm. The distribution area of points with maximum positioning errors using the two different training methods is almost the same. The positioning accuracy of Figure 7 and Figure 13 in the center area is lower than that of the outer edges. This is mainly due to fluctuations of the light intensity in this red region being noticeably larger. In fact, the fluctuation of the LEDs’ illumination is not consistent and will lead to an increase in positioning errors, according to the [23]. The maximum positioning error for the arbitrary training data set is obviously lower due to more training points in the area.

Then we compare the performance of our system with other localization algorithms which have been reported and the parameters are shown in Table 6.

**Figure 18:**
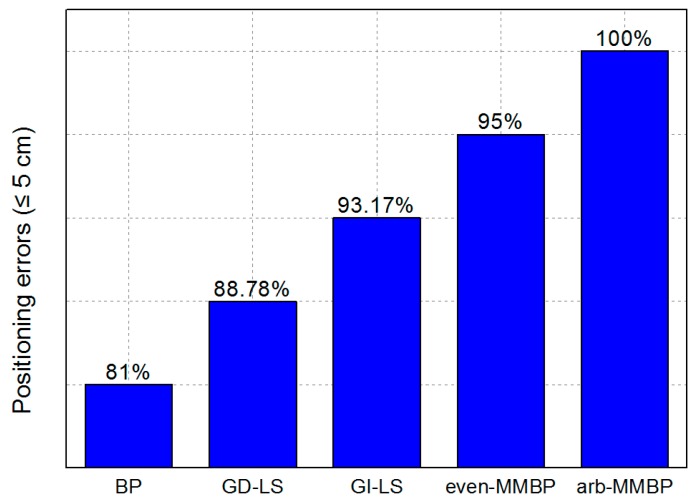
Probabilities of positioning errors less than 5 cm with different algorithms.

Comparison results of the probability of positioning errors being less than 5 cm using different algorithms are shown in Figure 18. Here we have used “arb-MMBP” to denote the MMBP trained by the arbitrary data set, and “even-MMBP” for that trained by the even data set. Comparing the performance of the five positioning systems, while the probability of positioning errors being less than 5 cm for BP, GD-LS, and GI-LS algorithms are only 81%, 88.78%, and 93.17%, respectively, that for the even-MMBP and arb-MMBP methods are as high as 95% and 100%. This suggests that our positioning accuracy is even better than the performance optimized by the GD-LS and GI-LS algorithms, which are verified by 225 training points utilizing the fingerprint base. It is to be noted that the size of our training set is only 20, which is 1/11 of that used for GD-LS and GI-LS in [15]. This is also the conclusion reached while comparing our results with [13], and the training set used in the BP method is 283 times larger than ours. In addition, the dimension of our positioning area is seven times larger than that in [15], and is the largest among the three systems. In a word, compared with the results in [13,15], we have achieved a higher positioning accuracy with the least training data in the largest positioning scenario. All of the above results indicate that the high-precision, easy-training and fast-convergent MMBP algorithm can be more widely adopted in practical scenarios. 

## 4. Conclusions

In this letter, a visible light positioning system using an RSS- and ANN-based MMBP algorithm is implemented in an indoor environment with four LEDs and a detector. Results indicate that the average positioning error with the MMBP algorithm is 1.88 cm for the arbitrary set and 1.99 cm for the even set with only 20 training points in the 1.8 m × 1.8 m × 2.1 m area. Compared with the traditional RSS localization algorithm, the average positioning accuracy of the MMBP algorithm is 7.6 times higher. It is also higher than many machine-learning-based reports of RSS and RSS fingerprint base algorithms. More importantly, in comparison with the other positioning algorithms mentioned in this paper, we have realized the highest positioning accuracy with the least number of training points in the largest localization area, which is the closest analog to real scenarios. In addition, the training and positioning durations for the even set are only 2.36 s and 0.007 s, respectively; while for the arbitrary set the two processes are sped up even more to 0.403 s and 0.005 s. The timeliness results show that the MMBP algorithm has a fast convergence rate. In conclusion, the MMBP algorithm proves to be highly precise, easily trained and rapidly convergent, demonstrating the potential to be widely used in actual indoor positioning scenarios.

## Figures and Tables

**Table 1:** Specific Parameters of Positioning System.

Parameters	Value
Injection current of LEDs (A)	1
Receiver active area diameter (mm)	1
Responsivity of detector (A/W)	25 (@600 nm)
Sampling rate of oscilloscope (MSa/s)	100
Half power angle of LEDs	45°
LED 3dB bandwidth	3 MHz

**Table 2:** Parameters of the neural network with the even training.

Parameter	Value
Hidden layer (L)	13
Learning rate (η)	0.0003
Momentum factor	0.9
Iteration times	6594
Constant increment factor ($k_{inc}$)	1.01
Constant decrement factor ($k_{dec}$)	0.75

**Table 3:** Time cost of different algorithms with even training data set.

Parameters	MMBP Algorithm	Traditional RSS-Based Algorithm
Training time (s)	2.36	NAN
Positioning time (s)	0.007	2.25

**Table 4:** Parameters of the neural network with the arbitrary training data set.

Parameter	Value
Hidden layer (L)	10
Learning rate (η)	0.001
Momentum factor (α)	0.9
Iteration times	975
Constant increment factor ($k_{inc}$)	1.065
Constant increment factor ($k_{dec}$)	0.4

**Table 5:** Time cost of different algorithms with arbitrary training data set.

Parameters	MMBP Algorithm	Traditional RSS-Based Algorithm
Training time (s)	0.403	NAN
Positioning time (s)	0.005	2.25

**Table 6:** Parameters of different location system.

	MMBP	BP [13]	GD-LS [15]	GI-LS [15]
Training points	20	5680	225	225
Location area	1.8 m × 1.8 m	1 m × 0.9 m	0.7 m × 0.7 m	0.7 m × 0.7 m
Positioning method	RSS	RSS	RSS-fingerprint base	RSS-fingerprint base

## References

[1] Xie Z., Guan W., Zheng J., Zhang X., Chen S., Chen B. (2019). A High-Precision, Real-Time, and Robust Indoor Visible Light Positioning Method Based on Mean Shift Algorithm and Unscented Kalman Filter. Sensors.

[2] Zhang R., Zhong W., Qian K., Wu D. (2017). Image Sensor Based Visible Light Positioning System With Improved Positioning Algorithm. IEEE Access.

[3] Şahin A., Eroğlu Y.S., Güvenç İ., Pala N., Yüksel M. (2015). Hybrid 3-D Localization for Visible Light Communication Systems. J. Lightwave Technol..

[4] Rahman M.S., Kim K.-D. (2013). Indoor Location Estimation Using Visible Light Communication and Image Sensors. Int. J. Smart Home.

[5] Zhang W., Kavehrad M. A 2-D indoor localization system based on visible light LED. Proceedings of the 2012 IEEE Photonics Society Summer Topical Meeting Series.

[6] Zheng H., Xu Z., Yu C., Gurusamy M. Indoor three-dimensional positioning based on visible light communication using Hamming filter. Proceedings of the Advanced Photonics Congress 2016.

[7] Yang A., Feng L., Liu X., Feng L. (2015). Combination of light-emitting diode positioning identification and time-division multiplexing scheme for indoor location-based service. Chin. Opt. Lett..

[8] Do T.-H., Yoo M. (2016). An in-Depth Survey of Visible Light Communication Based Positioning Systems. Sensors.

[9] Lee S.J., Yoo J.H., Jung S.Y. VLC-based indoor location awareness using LED light and image sensors. Proceedings of the 2012 Photonics Asia.

[10] Hossen M.S., Park Y., Kim K.D. (2015). Performance improvement of indoor positioning using light-emitting diodes and an image sensor for light-emitting diode communication. Opt. Eng..

[11] Fu M., Zhu W., Le Z., Manko D., Gorbov I., Beliak I. (2017). Weighted average indoor positioning algorithm that uses LEDs and image sensors. Photonic Netw. Commun..

[12] Huang H.Q., Yang A.Y., Feng L.H., Ni G.Q., Guo P. (2017). Artificial neural-network-based visible light positioning algorithm with a diffuse optical channel. Chin. Opt. Lett..

[13] Hsu C.-W., Liu S.M., Lu F., Chow C.-W., Yeh C.-H., Chang G.-K. Accurate Indoor Visible Light Positioning System utilizing Machine Learning Technique with Height Tolerance. Proceedings of the 2018 Optical Fiber Communications Conference and Exposition (OFC).

[14] Chen Z., Wang J. (2018). GROF: Indoor Localization Using a Multiple-Bandwidth General Regression Neural Network and Outlier Filter. Sensors.

[15] Guo X., Shao S., Ansari N., Khreishah A. (2017). Indoor Localization Using Visible Light Via Fusion of Multiple Classifiers. IEEE Photonics J..

[16] Guo X., Hu F., Elikplim N.R., Li L. (2019). Indoor Localization Using Visible Light via Two-Layer Fusion Network. IEEE Access.

[17] Horikawa S., Furuhashi T., Uchikawa Y. (1992). On fuzzy modeling using fuzzy neural networks with the back-propagation algorithm. IEEE Trans. Neural Networks.

[18] Miao K., Chen Y., Miao X. An indoor positioning technology based on GA-BP Neural Network. Proceedings of the 2011 6th International Conference on Computer Science & Education (ICCSE).

[19] Wang C., Wu F., Shi Z., Zhang D. (2016). Indoor positioning technique by combining RFID and particle swarm optimization-based back propagation neural network. Optik.

[20] Mehmood H., Tripathi N.K. (2013). Optimizing artificial neural network-based indoor positioning system using genetic algorithm. Int. J. Digital Earth.

[21] Guan W., Wu Y., Xie C., Chen H., Cai Y., Chen Y. (2017). High-precision approach to localization scheme of visible light communication based on artificial neural networks and modified genetic algorithms. Opt. Eng..

[22] Rumelhart D.E., Hinton G.E., Williams R.J. (1986). Learning representations by back-propagating errors. Nature.

[23] Lv H., Feng L., Yang A., Guo P., Huang H., Chen S. (2017). High Accuracy VLC Indoor Positioning System With Differential Detection. IEEE Photonics J..

